# Bioactive Compound Recovery from Apple Pomace by Aqueous Ultrasound-Assisted Extraction: Machine Learning Modelling and Multi-Objective Optimization

**DOI:** 10.3390/antiox15091215

**Published:** 2026-09-21

**Authors:** Biljana Lončar, Milena Terzić, Aleksandra Cvetanović Kljakić, Mirjana Petronijević, Sanja Panić, Jelena Arsenijević, Gokhan Zengin, Slavica Ražić

**Affiliations:** 1 Faculty of Technology Novi Sad, University of Novi Sad, Bulevar cara Lazara 1, 21000 Novi Sad, Serbia; biljanacurcicc@gmail.com (B.L.); a.c.istrazivac@gmail.com (A.C.K.); mirjana.petronijevic@uns.ac.rs (M.P.); sanjar@tf.uns.ac.rs (S.P.); 2 Faculty of Pharmacy, University of Belgrade, Vojvode Stepe 450, 11000 Belgrade, Serbia; jelena.arsenijevic@pharmacy.bg.ac.rs (J.A.); slavica.razic@pharmacy.bg.ac.rs (S.R.); 3 Science Faculty, Selcuk University, Campus Konya, Konya 42130, Turkey; biyologzengin@gmail.com

**Keywords:** apple pomace, aqueous ultrasound-assisted extraction, phenolic compounds, antioxidant activity, enzyme inhibition, machine learning, process optimization, sustainable valorisation

## Abstract

Apple pomace represents a sustainable source of phenolic compounds with significant antioxidant potential. This study investigated the recovery of water-extractable bioactive constituents from apple pomace using aqueous ultrasound-assisted extraction (UAE) combined with biochemical profiling and machine learning-based modelling. Extraction conditions were varied according to time (10–30 min), temperature (25–75 °C), and solvent-to-solid ratio (10–20 mL/g). The obtained extracts were evaluated for total phenolic content (TP), total flavonoid content (TF), antioxidant capacity (DPPH, ABTS, CUPRAC, FRAP, metal chelating, and phosphomolybdenum assays), and enzyme inhibitory activities against acetylcholinesterase, butyrylcholinesterase, tyrosinase, α-amylase, and α-glucosidase. TP and TF ranged from 4.70 to 9.97 mg GAE/g and 0.21–0.79 mg RE/g, respectively, while antioxidant assays demonstrated substantial variation depending on extraction conditions. Strong correlations (r = 0.827–0.945, *p* < 0.001) were observed between phenolic content and antioxidant activity. Artificial neural networks, random forests, support vector machines, and a hybrid ensemble model were applied to predict extraction outcomes, with predictive performance varying substantially among biochemical responses and modelling approaches. Multi-objective optimization using the NSGA-II formulation identified a representative Pareto compromise at approximately 15.1 min, 25.7 °C, and a solvent-to-solid ratio of 13.7 mL/g, balancing desirable biochemical responses with processing requirements.

## 1. Introduction

Apples (*Malus domestica*) rank among the most extensively cultivated and consumed fruits globally, due to their favourable sensory attributes, high nutritional value, and associated health benefits. They are rich in dietary fibre, vitamins, and a wide spectrum of polyphenolic compounds, including flavonoids and phenolic acids, with well-documented antioxidant, anti-inflammatory, cardioprotective, and neuroprotective activities [1,2,3]. Worldwide apple production consistently exceeds ~89 million tonnes, with an average per capita availability of approximately 10 kg annually, while the global market for apple and apple-derived products is estimated at ~90–95 billion USD, reflecting strong and sustained consumer demand for both fresh fruit and processed commodities such as juices, purees, and functional ingredients [4].

In the Republic of Serbia, apple cultivation constitutes a cornerstone of horticultural agriculture, with harvested areas of around 26,000–27,000 ha and annual production in the hundreds of thousands of tonnes, reflecting its economic importance within the national fruit sector [5,6]. A substantial share of this output supplies the domestic market, while significant volumes are exported to markets in the European Union, the Middle East, and beyond, contributing to agricultural revenue and supporting rural economies [7]. Despite this robust production and trade, industrial processing of apples generates large quantities of solid residues, commonly referred to as apple pomace [8]. Depending on pressing and extraction methods, pomace can account for 10–35% of the fresh fruit mass, amounting to millions of tonnes of agro-industrial by-products on a global scale [9]. Due to high moisture content and rapid spoilage, apple pomace is predominantly relegated to low-value uses such as animal feed, composting, or landfill disposal, representing a significant waste management challenge and an underutilized resource [10].

Apple pomace contains appreciable levels of bioactive phytochemicals, including phenolic acids (e.g., chlorogenic acid), flavonoids (e.g., quercetin derivatives and phloridzin), and dietary fibres, which are associated with health-promoting properties [11]. Recovering these compounds through efficient extraction technologies aligns with sustainable valorisation strategies and the emerging demand for natural functional ingredients [12]. Ultrasound-assisted extraction (UAE) has emerged as a promising green technology for this purpose, enhancing mass transfer and disrupting plant cell matrices to achieve higher yields of target compounds with reduced solvent consumption and processing times compared to conventional methods. The application of UAE to apple pomace has demonstrated effective recovery of phenolic fractions with significant antioxidant potential [13,14,15,16,17,18].

The optimization of UAE, however, remains complex due to nonlinear interactions among key process parameters, such as extraction time, temperature, and solvent-to-solid ratio, and multiple functional responses, including total phenolic content and antioxidant activity [19,20]. Advanced machine learning (ML) approaches offer a powerful strategy to address this complexity; algorithms such as artificial neural networks (ANNs), random forests (RF), and support vector machines (SVMs) can model nonlinear relationships in experimental data, enabling exploratory prediction of extraction outcomes and supporting the identification of favourable process conditions within the investigated experimental domain [21,22].

Despite the growing interest in ultrasound-assisted extraction of phenolic compounds from apple pomace, existing studies predominantly rely on conventional optimization approaches and focus on limited response variables, while the integration of multi-response biochemical profiling with advanced machine learning-driven predictive modelling and multi-objective optimization remains largely unexplored. In particular, previous studies rarely integrate multi-response biochemical characterization (antioxidant and enzyme-inhibitory activities), multiple machine learning algorithms, and evolutionary multi-objective optimization within a single unified framework.

Therefore, this study proposes an integrated data-driven approach that combines experimental design, multi-target biochemical profiling, and ensemble machine learning modelling coupled with NSGA-II optimization. Unlike conventional response surface methodology (RSM)-based studies, this framework enables simultaneous modelling of complex nonlinear relationships and optimization across multiple, potentially conflicting objectives.

The novelty of this work lies in the simultaneous coupling of: (i) aqueous ultrasound-assisted extraction as a green technology, (ii) multi-output machine learning prediction (ANN, RF, SVM, and hybrid ensemble), and (iii) Pareto-based multi-objective optimization, providing a more comprehensive strategy for the valorisation of apple pomace into functional bioactive ingredients. Accordingly, the present study focuses on the optimization of process parameters within a defined aqueous UAE system, with solvent composition and phenolic subclass-specific recovery remaining outside the scope of the experimental design.

## 2. Materials and Methods

### 2.1. Chemicals and Reagents

All chemicals used for the analysis were of analytical grade and obtained from Sigma-Aldrich (Darmstadt, Germany), unless otherwise stated. The reagents included 2,2′-azino-bis(3-ethylbenzothiazoline-6-sulphonic acid (ABTS), 1,1-diphenyl-2-picrylhydrazyl (DPPH), gallic acid, rutin, electric eel acetylcholinesterase (AChE) (type-VI-S, EC 3.1.1.7), horse serum butyrylcholinesterase (BChE) (EC 3.1.1.8), galantamine, acetylthiocholine iodide (ATChI), butyrylthiocholine chloride (BTChI), 5,5-dithio-bis(2-nitrobenzoic) acid (DTNB), tyrosinase (EC 1.14.18.1, mushroom), glucosidase (EC 3.2.1.20, from *Saccharomyces cerevisiae*), amylase (EC 3.2.1.1, from porcine pancreas), sodium molybdate, sodium nitrate, sodium carbonate, hydrochloric acid, Trolox, ethylenediaminetetraacetate (EDTA), neocuproine, cupric chloride, ammonium acetate, ferric chloride, 2,4,6-tris(2-pyridyl)-s-triazine (TPTZ), ammonium molybdate, ferrozine, ferrous sulphate hexahydrate, kojic acid, and acarbose. Folin–Ciocalteu reagent (cat. no. F9252) and aluminium chloride (AlCl_3_, cat. no. 11019) were purchased from Merck (Darmstadt, Germany).

### 2.2. Material

The apple pomace used in this study was supplied by Nectar d.o.o. (Bačka Palanka, Serbia) as a by-product fruit processing facility. Afterwards, it was transported to the laboratory and subjected to analysis. The obtained apple pomace samples were frozen and stored at −30 °C for 24 h, then subjected to lyophilization (Christ ALPHA 1–2 LD PLUS, Osterode am Harz, Germany). The lyophilization parameters were set as follows: pressure of 1.6 Pa, condenser temperature of −57 °C, shelf temperature at room temperature, and process duration of 48 h. These lyophilization conditions were selected based on the previously established procedure [23] to ensure adequate dehydration of the apple pomace while limiting thermal exposure and preserving thermolabile bioactive constituents prior to extraction. After lyophilization, the samples were finely ground into a powder using a universal laboratory mill, type WZ-1 (Solem, ZBPP, Bydgoszcz, Poland).

### 2.3. Ultrasound-Assisted Extraction (UAE)

Apple pomace was extracted using water as the sole extraction solvent. Water was selected a priori as a food-compatible and environmentally preferable extraction medium; therefore, solvent composition was not included as an optimization variable. The purpose of the experimental design was to optimize the process conditions of aqueous UAE rather than to identify the optimal solvent composition for individual phenolic subclasses. For each extraction, 5.0 g of lyophilized and ground apple pomace was transferred into a 150 mL Erlenmeyer flask, and 50, 75, or 100 mL of water was added to obtain the solvent-to-solid ratios of 10, 15, and 20 mL/g, respectively, as specified in Table 1. Extraction was performed in an ultrasonic bath (Elma Schmidbauer GmbH, Konstanz, Germany) operating at 45 kHz and 50% ultrasonic power. Extraction time (10–30 min), temperature (25–75 °C), and solvent-to-solid ratio (10–20 mL/g) were varied according to the experimental matrix presented in Table 1. During extraction, the flasks were fitted with ground-glass stoppers and additionally sealed with parafilm to minimize solvent evaporation. After extraction, the crude extracts were filtered through 120 mm Whatman^®^ filter paper and collected in glass vials without subsequent adjustment to a fixed final volume. The resulting aqueous extracts were used directly in liquid form for subsequent biochemical analyses without evaporation or drying. The final recovered filtrate volume was not independently measured after filtration; therefore, the stated volumes refer to the initial solvent volumes used for extraction. Gravimetric extraction yield could not be determined, as the aqueous extracts were neither evaporated nor dried to constant mass. Therefore, the reported biochemical responses were normalized to the initial dry mass of apple pomace rather than to the mass of recovered dry extract. This should be considered when comparing the present results with studies reporting concentrations or activities on a dry-extract basis. Each extraction condition was independently performed in triplicate, and the resulting extracts were stored at 4 °C until analysis.

The experimental matrix represents a structured multi-factor variation designed to cover the selected parameter space (time, temperature, and solvent-to-solid ratio), rather than a formal response surface design (Box–Behnken or central composite design). This approach was selected to ensure sufficient variability for subsequent machine learning modelling while maintaining a manageable number of experimental runs. However, it should be noted that the absence of a formal design of experiments (DOE) may limit the statistical interpretability of factor interactions.

### 2.4. Greenness Metric for Sample Preparation

**Figure 1:**
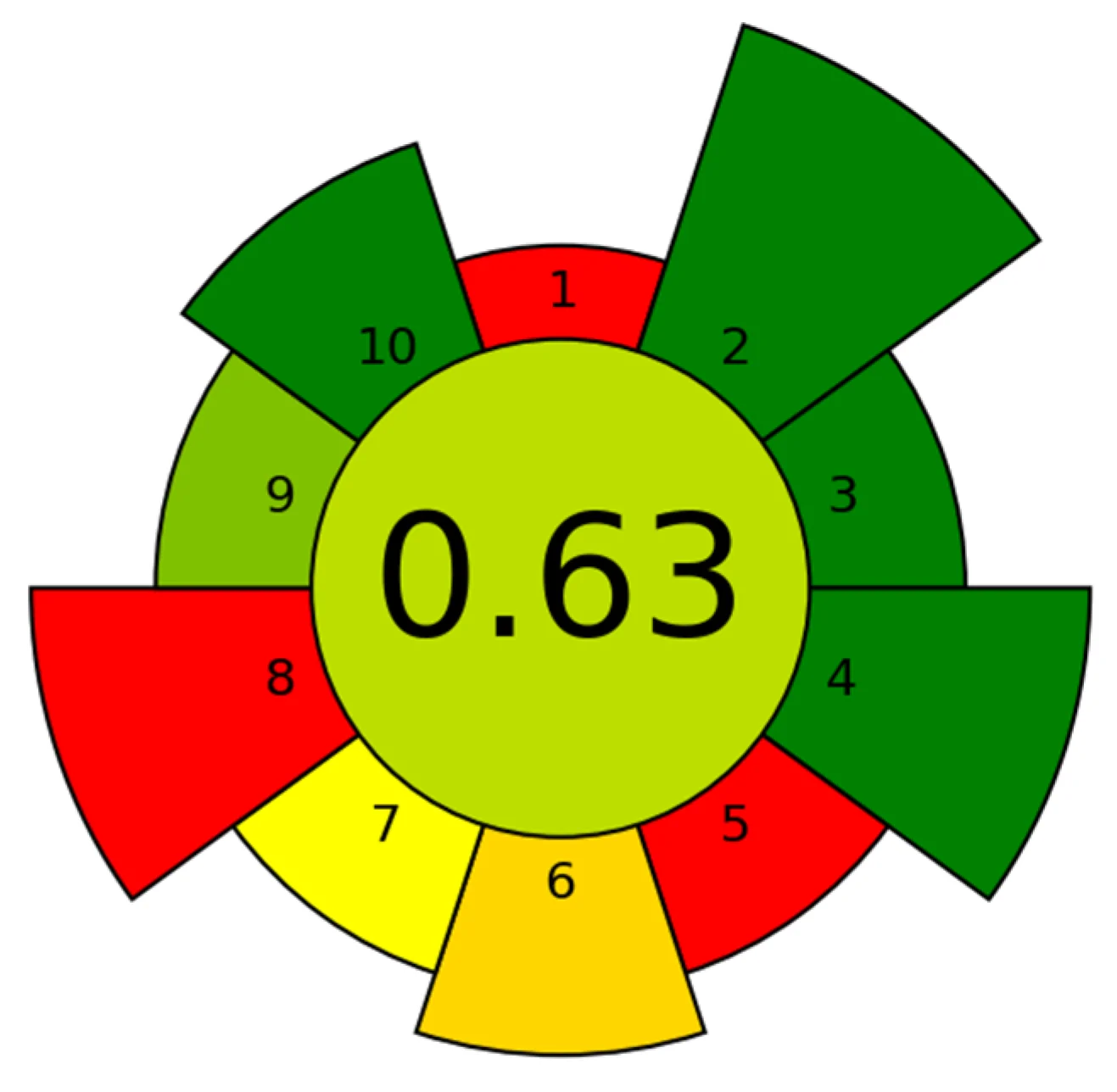
Greenness assessment of the apple pomace extracts preparation—AGREEprep pictogram.

Sample preparation remains a bottleneck in the method selected for measuring either a specific analyte or bioactivity. It is not sufficient to declare that a method, procedure, or solvent is “green.” Analytical tools for green metrics are available, and each step in sample preparation should be carefully assessed and related to established principles. The ten principles of green sample preparation provide excellent guidance to evaluate procedures and determine how truly green our preparation process is [24]. Based on this framework, AGREEprep was developed as a metric tool [25]. The results are expressed as sub-scores and a final score on a scale from 0 to 1. Not only are hazardous materials, solvent selection, sample size and throughput, waste, energy consumption, sustainability, and reusability assessed, but the weights for all criteria are also assessed. The application of the AGREEprep green metric to our sample preparation procedure resulted in a final score of 0.63, corresponding to moderate greenness (Figure 1).

Compared to conventional solvent extraction methods, the use of water as an extraction solvent combined with ultrasound assistance contributes to reduced environmental impact. However, it should be noted that ultrasound processes involve energy consumption, which should be considered when evaluating overall process sustainability at an industrial scale. The obtained AGREEprep score (0.63) indicates moderate greenness, suggesting that while the method aligns with several green chemistry principles, there is still potential for further optimization.

### 2.5. Determination of Total Phenolic (TP) and Flavonoid Content (TF)

The content of total phenolics in apple pomace extracts was estimated spectrophoto-metrically using the Folin–Ciocalteu reagent (Sigma-Aldrich, St. Louis, MO, USA), according to the method described by [26]. The results were normalized to the initial dry mass of lyophilized apple pomace used for extraction and were expressed as mg gallic acid equivalents (GAE) per g dry apple pom-ace. Total flavonoids were quantified by applying the aluminium chloride (AlCl_3_) colorimetric assay according to the procedure reported by [26]. The results were expressed as mg rutin equivalents (RE) per g dry apple pomace.

### 2.6. Antioxidant and Enzyme Inhibitor Activity

The antioxidant properties of the obtained extracts were investigated using several complementary in vitro assays, including DPPH and ABTS radical scavenging methods, as well as CUPRAC, FRAP, metal chelating (MH), and phosphomolybdenum (PM) assays [19,26]. The antioxidant capacities determined by the DPPH, ABTS, CUPRAC, and FRAP methods were expressed as mg Trolox equivalents (TE) per g dry apple pomace. The metal chelating ability was reported as mg EDTA equivalents (EDTAE) per g dry apple pomace, whereas the results of the phosphomolybdenum assay were expressed as mmol TE per g dry apple pomace.

Enzyme inhibitory potential was evaluated against acetylcholinesterase (AChE), butyrylcholinesterase (BChE), tyrosinase, α-amylase, and α-glucosidase. Galanthamine served as a reference inhibitor for cholinesterases, kojic acid for tyrosinase, and acarbose for carbohydrate-hydrolysing enzymes. The inhibitory activities were expressed as galanthamine equivalents (GALAEs), kojic acid equivalents (KAEs), and acarbose equivalents (ACAEs) per g dry apple pomace, respectively [27]. All measurements were carried out in triplicate, and the results were expressed as mean ± standard deviation (SD).

### 2.7. Statistical Analysis

Statistical processing of the obtained experimental data was conducted using StatSoft Statistica 10.0^®^ software. The distribution of the data was assessed using the Shapiro–Wilk test, while homogeneity of variances among experimental conditions was evaluated using Levene’s test. Because significant deviations from normality were observed for several response variables, differences among the 15 experimental conditions were additionally evaluated using the non-parametric Kruskal–Wallis test, followed, where appropriate, by Dunn’s post hoc test with Holm correction for multiple comparisons. One-way analysis of variance (ANOVA) followed by Tukey’s honestly significant difference (HSD) test was used for the parametric comparison of mean values reported in Table 2, Table 3 and Table 4. Statistical significance was considered at *p* < 0.05. For graphical visualization of the dataset, a colour correlation analysis plot diagram and hierarchical cluster analysis were generated using ChiPlot (https://www.chiplot.online).

To enable an integrated comparison of the biochemical performance of the experimentally obtained extracts, all measured biochemical responses were transformed to dimensionless standardized scores using min–max normalization. Since higher values of TP, TF, antioxidant responses (DPPH, ABTS, CUPRAC, FRAP, MH, and PM), and enzyme-inhibitory activities (AChE, BChE, tyrosinase, α-amylase, and α-glucosidase) were considered desirable in the context of the present biochemical screening, a larger-the-better criterion was applied:

(1) $$S_{ij}=\left(x_{ij}-x_{j,min}\right)/\left(x_{j,max}-x_{j,min}\right)$$

where S_ij_ is the standardized score of response j for experimental condition i, x_ij_ is the corresponding experimental value, and x_j,min_ and x_j,max_ are the minimum and maximum experimental values observed for response _j_, respectively. An overall biochemical performance score was subsequently calculated as the arithmetic mean of the 13 individual standardized scores, with equal weighting assigned to all responses.

### 2.8. Mathematical Models Development

The modelling workflow was implemented in R ver. 4.4.3, using a combination of neural networks (ANNs) [28,29,30], random forests (RFs) [31], support vector machines (SVMs) [32], and a hybrid model [33]. A hybrid predictive model was constructed by integrating the outputs of the three individually trained machine learning models: ANN, RF, and SVM, in order to improve predictive accuracy and reduce model-specific variability [29,34]. Each model was first calibrated using the same training dataset [35], after which predictions were generated independently for both the training and test subsets. The hybrid prediction for each observation was obtained by computing the arithmetic mean of the three model outputs [36,37], thereby forming a soft-voting ensemble that combines the nonlinear learning capability of the ANN model, the variance-reducing properties of the RF model, and the boundary-optimizing characteristics of the SVM model [38]. By averaging the predictions of the individual models, the hybrid approach was intended to reduce model-specific variability and provide a more balanced ensemble prediction [39]. Its predictive performance was subsequently evaluated against the individual models using the same LOOCV framework.

Four models were used to predict the extraction outcomes of apple pomace based on three key process variables, which served as independent variables: extraction time, extraction temperature, and solvent-to-solid ratio.

The responses modelled included total phenolics (TPs), total flavonoids (TFs), multiple antioxidant assays (DPPH, ABTS, CUPRAC, and FRAP), and enzyme inhibition activities (MH, PM, AChE, BChE, tyrosinase, α-amylase, and α-glucosidase), representing the multifunctional bioactivity profile of apple pomace extracts.

To evaluate model performance, the experimental dataset comprised 15 independent extraction conditions, with each response measured in triplicate. Given the limited number of independent experimental conditions, model validation was performed using leave-one-condition-out cross-validation (LOOCV) [40]. In each of the 15 iterations, all three replicate measurements corresponding to one experimental condition were excluded from model fitting and used as the test set, whereas the replicate measurements from the remaining 14 experimental conditions were used for model training. The procedure was repeated until each experimental condition had served once as the held-out test condition. Replicate measurements belonging to the same experimental condition were always kept together within either the training or the test subset to prevent information leakage between model fitting and evaluation. Performance metrics obtained across the LOOCV iterations were subsequently aggregated to provide an overall assessment of model performance.

Model performance was evaluated using the coefficient of determination (R^2^), root mean square error (RMSE), sum of squared errors (SSEs), and average absolute relative deviation (AARD) [28,41]. The coefficient of determination was calculated according to Equation (2):

(2) $$R=1-\frac{\sum_{i=1}^{n}{\left(y_{i}-{\hat{y}}_{i}\right)}^{2}}{\sum_{i=1}^{n}{\left(y_{i}-{\bar{y}}_{i}\right)}^{2}}$$

where y is the experimentally determined value, ${\hat{y}}_{i}$ is the corresponding model-predicted value, and ${\bar{y}}_{i}$ is the mean of the experimental values. Negative R^2^ values were retained in the analysis because they indicate that the corresponding model exhibited poorer predictive performance than prediction based on the response mean. Given the limited number of independent experimental conditions, the resulting LOOCV performance metrics were interpreted as exploratory estimates of predictive capability within the investigated experimental domain rather than as evidence of broad model generalizability.

Following model validation, the developed models were subsequently incorporated into the multi-objective optimization procedure [42,43] using the mco package to identify extraction conditions that jointly maximize phenolic content, antioxidant capacity, and enzyme-inhibitory activities while minimizing process-intensity variables. This design ensures that the modelling framework systematically evaluates the predictive relationships between extraction conditions and the resulting biochemical responses while reducing the dependence of performance estimates on a single train–test partition.

## 3. Results

### 3.1. Statistical Evaluation of Experimental Responses

Preliminary distributional analysis showed that several measured responses deviated significantly from normality. Shapiro–Wilk testing indicated no significant departure from normality for DPPH, ABTS, PM, AChE, and BChE (*p* > 0.05), whereas significant deviations were detected for several other responses. After false-discovery-rate correction, evidence of non-normality remained significant for TP, CUPRAC, FRAP, MH, tyrosinase, α-amylase, and α-glucosidase. Levene’s test indicated homogeneity of variances among experimental conditions for all responses except TP (*p* = 0.015), while α-amylase (*p* = 0.070) and α-glucosidase (*p* = 0.064) showed borderline variance heterogeneity.

Considering these distributional characteristics, the differences among the 15 experimental conditions were additionally examined using the Kruskal–Wallis test. Significant differences among conditions were observed for all 13 measured responses (*p* ≤ 0.00153), with large effect sizes (ε^2^ = 0.696–0.974). The strongest effects were observed for CUPRAC (ε^2^ = 0.974), ABTS (ε^2^ = 0.972), FRAP (ε^2^ = 0.935), and TP (ε^2^ = 0.930). All Kruskal–Wallis *p*-values remained significant after Holm and false-discovery-rate corrections, confirming robust overall differences among the investigated extraction conditions.

### 3.2. Total Phenolic and Flavonoid Content

The total phenolic content (TP) and total flavonoid content (TF) of apple pomace extracts under different ultrasound-assisted extraction (UAE) conditions are presented in Table 2. TP values ranged from 4.70 to 9.97 mg GAE/g, while TF values ranged from 0.21 to 0.79 mg RE/g. Total polyphenol values reported in the literature for apple pomace vary widely depending on the extraction method, solvent, and cultivar, generally ranging from approximately 5 to 22 mg GAE/g [9,44,45], while total flavonoid content shows similarly broad variation [44]. The TP values obtained in the present study fall within or near the lower boundary of this reported range, which is consistent with the use of water as the sole extraction solvent. Aqueous systems generally afford lower phenolic recovery compared with hydroalcoholic mixtures due to differences in solvent polarity and the reduced solubilisation capacity for moderately lipophilic phenolic compounds. Aqueous-methanol extraction of apple pomace has been reported to yield TP values of 5.56 mg GAE/g [44], while subcritical water extraction under optimised conditions yielded substantially higher values of 39.08 mg GAE/g [45,46], highlighting the strong influence of extraction method and solvent system on phenolic recovery. The notably lower TF values obtained in the present study compared with literature reports (14.3–25.0 mg RE/g) similarly reflect the aqueous extraction system employed, as some flavonoid subclasses may exhibit lower aqueous extractability than more hydrophilic phenolic constituents. However, chlorogenic acid, reported as a major phenolic constituent of apple pomace [47], is highly water-soluble due to its ester-linked quinic acid moiety, which may partly contribute to the comparatively more favourable TP recovery relative to TF under the aqueous conditions applied in this study.

The highest TP values were observed in samples 11 and 12, which were not significantly different from each other (Table 2). The high TP obtained for sample 11 may be associated with enhanced mass transfer and acoustic cavitation-driven cell wall disruption, which are known to facilitate the release of phenolics from plant matrices [48]. Studies on UAE of apple pomace have confirmed that temperature and ultrasound amplitude are the dominant parameters governing phenolic recovery, with maximum phenolic extraction achieved at 90 °C and 50% ultrasound amplitude using water as solvent [15]. Although the temperature associated with the highest TP under the investigated experimental conditions (75 °C) was somewhat lower, this difference is likely attributable to variations in solid-to-liquid ratio, ultrasound frequency, and material pre-treatment between studies. Sample 12, extracted at low temperature (25 °C) with a high solvent-to-solid ratio (20 mL/g), achieved a comparable TP value through a different combination of extraction conditions. A higher solvent-to-solid ratio modifies the solubility and equilibrium constants, thereby potentially promoting phenolic recovery even under lower thermal input [49]. The favourable performance of sample 12 may be associated with the higher solvent-to-solid ratio, which can promote mass transfer by maintaining a favourable concentration gradient between the solid matrix and the extraction medium [49]. This suggests that a favourable mass-transfer gradient may partly compensate for lower thermal energy input under the investigated conditions.

Samples 8 and 9 also showed above-average TP and TF values, suggesting that moderate thermal input combined with adequate solvent availability may provide favourable phenolic recovery under the investigated conditions. The TP values recorded for these mid-range samples (5–7 mg GAE/g) are in good agreement with aqueous UAE studies on apple pomace reporting values between 2.23 and 6.18 mg GAE/g [15], further corroborating the validity of the results obtained in the present study.

Samples obtained under less favourable combinations of solvent-to-solid ratio, temperature, and extraction time generally yielded lower TP and TF values, likely reflecting less efficient mass transfer and incomplete disruption of the plant cell wall matrix. Notably, sample 15, extracted at 75 °C for 30 min and at a high solvent-to-solid ratio (20 mL/g), yielded the lowest TP (4.70 mg GAE/g), despite the same temperature in sample 11 producing the highest phenolic content. This finding indicates that phenolic recovery was governed by the combined effects of extraction temperature, time, and solvent availability rather than by temperature alone [15,50].

Within the investigated combinations of extraction conditions, variations associated with extraction time appeared less pronounced than those observed across conditions differing in temperature and solvent-to-solid ratio; however, their independent contribution cannot be separated from the simultaneous variation of the other process factors. The dominant phenolic compounds in apple pomace—chlorogenic acid, quercetin glycosides, and phloridzin [48,51]—differ considerably in their aqueous solubility and diffusivity, which means that extraction conditions favouring the recovery of hydrophilic phenolic acids do not necessarily promote the simultaneous extraction of less polar flavonoid compounds. This likely accounts for the observed differences in TP and TF responses across samples under varying extraction conditions. Overall, the observed variation in phenolic and flavonoid recovery reflected the combined influence of extraction time, temperature, and solvent-to-solid ratio. Because the experimental matrix was designed as a structured multi-factor variation rather than a one-variable-at-a-time or formal response-surface design, the independent contribution of each extraction factor cannot be unequivocally isolated from these data. Nevertheless, comparisons among selected conditions suggest that temperature and solvent availability contributed substantially to the observed differences in TP and TF, consistent with previously reported findings for UAE of apple pomace [15].

### 3.3. Antioxidant Activity of Apple Pomace Extracts

The antioxidant capacity of apple pomace extracts under different ultrasound-assisted extraction (UAE) conditions was assessed using six complementary assays, DPPH, ABTS, CUPRAC, FRAP, metal chelation (MH), and phosphomolybdenum (PM), providing a multi-mechanistic evaluation of their radical scavenging, reducing, and metal-binding potential (Table 3). The use of multiple assays was methodologically warranted, as different assay principles respond selectively to different structural features of antioxidant molecules [50].

DPPH and ABTS radical scavenging capacities ranged from 11.17 to 15.51 mg TE/g and from 7.28 to 16.84 mg TE/g, respectively. These values are notably higher than those reported for aqueous apple pomace extracts [52,53], which is consistent with the use of UAE and lyophilised material in the present study, both of which are known to enhance phenolic recovery compared with conventional aqueous extraction of fresh pomace. The highest DPPH and ABTS values were observed in samples 11 and 12, which were not significantly different from each other (Table 3). The strong positive relationship between total phenolic content and DPPH and ABTS values observed across all samples suggests that the radical scavenging capacity of the extracts was primarily driven by their phenolic composition, consistent with previously reported correlations in apple-derived extracts [50,53], where procyanidin B2, chlorogenic acid, epicatechin, and quercetin have been identified as the dominant contributors to these activities [51].

CUPRAC values showed the widest variation of all assays (15.42–27.02 mg TE/g), with sample 11 displaying exceptionally high reducing power. The CUPRAC assay is particularly sensitive to phenolic compounds with strong electron-donating capacity, and the markedly higher CUPRAC value of sample 11 compared with sample 12 (27.02 vs. 22.96 mg TE/g) suggests that high-temperature extraction may have differentially enhanced the recovery of phenolic constituents contributing to CUPRAC reducing capacity [52]. FRAP values followed parallel trends (8.99–17.12 mg TE/g), with samples 11 and 12 again ranking highest, further confirming the superior redox-active phenolic content of these extracts. The consistent positive relationship between total phenolic content and both CUPRAC and FRAP values across all samples is in agreement with previously reported findings for apple-derived extracts [50].

Samples 8 and 9, extracted at intermediate temperature (50 °C) with moderate solvent ratios, also demonstrated above-average performance across all four assays, consistent with their above-average TP values reported in Section 3.1. This finding reinforces the conclusion that a balanced combination of moderate thermal input and adequate solvent availability is sufficient for satisfactory antioxidant recovery, and that elevated temperature is not a prerequisite for high antioxidant capacity [15,53].

The metal chelating activity ranged from 4.96 to 9.59 mg EDTAE/g, with sample 12 achieving the strongest chelating ability. The metal chelating capacity of phenolic compounds is primarily associated with the presence of ortho-dihydroxy (catechol) or galloyl groups, which form stable complexes with pro-oxidant transition metal ions such as Fe^2+^ and Cu^2+^ [54,55]. The superior chelating performance of sample 12 may indicate that the applied low-temperature, high-solvent-volume conditions favoured the recovery of compounds contributing to metal-chelating activity [55]. Notably, sample 11 demonstrated moderate metal chelating activity despite its superior performance in all other assays, indicating that high-temperature extraction differentially affects the recovery of compounds relevant to radical scavenging versus metal binding.

Phosphomolybdenum (PM) total antioxidant capacity ranged from 0.33 to 0.81 mmol TE/g. The PM assay responds broadly to both phenolic and non-phenolic antioxidant compounds, including vitamin E and ascorbic acid [56], which distinguishes it mechanistically from the other assays employed. Unexpectedly, sample 12 recorded the lowest PM value despite its superior performance in all other assays, likely reflecting limited co-extraction of lipophilic antioxidant compounds under high-dilution, low-temperature conditions. The contrasting high PM values of samples 1 and 4, despite their modest performance in other assays, further support this interpretation, suggesting that PM-active compounds in apple pomace are preferentially extracted under conditions suboptimal for total phenolic recovery.

Taken together, the six complementary assays revealed that UAE conditions exert complex, assay-dependent effects on the antioxidant profile of apple pomace extracts. Samples 11 and 12 consistently demonstrated the strongest overall antioxidant potential, reflecting their superior phenolic content, while the divergent MH and PM profiles across samples highlight the chemical complexity of apple pomace, further underscoring that no single assay is sufficient to fully characterise the antioxidant capacity of plant-derived extracts.

### 3.4. Enzyme Inhibitor Activity of Apple Pomace Extracts

The enzyme inhibitory activities of apple pomace extracts obtained under different UAE conditions are summarized in Table 4. All extracts exhibited measurable inhibitory activity against all five tested enzymes, AChE, BChE, tyrosinase, α-amylase, and α-glucosidase, with the magnitude of inhibition varying in a clearly condition-dependent manner, reflecting the sensitivity of different bioactive compound classes to extraction parameter settings. To the best of the authors’ knowledge, no previous study has systematically investigated the effect of UAE parameters on the enzyme inhibitory profile of aqueous apple pomace extracts across five biologically relevant targets simultaneously, making the present findings a novel contribution to the valorisation of this by-product.

Cholinesterase inhibition. AChE inhibition ranged from 2.08 to 2.58 mg GALAE/g, while BChE inhibition showed a broader range of 2.09 to 2.99 mg GALAE/g. AChE and BChE are the key enzymes involved in the hydrolysis of the neurotransmitter acetylcholine, and their inhibition is a primary pharmacological strategy in the management of Alzheimer’s disease [57,58]. The highest AChE inhibitory activity was recorded for samples 11 and 12, consistent with their elevated total phenolic content. Chlorogenic acid and quercetin, both characteristic phenolic compounds of apple pomace according to previously reported phytochemical profiles [51], have been identified as AChE inhibitors [59], and the higher total phenolic content of samples 11 and 12 likely reflects a greater overall abundance of such bioactive constituents, which may account for their superior AChE inhibitory activity. The cholinesterase inhibitory activity observed in the present study is consistent with previously reported findings for Annurca apple polyphenol extracts [59], which demonstrated concentration-dependent AChE and BChE inhibitory activity, with a slight preference for AChE, a selectivity pattern also reflected in the present study, where AChE inhibition showed narrower overall variation and stronger condition dependence than BChE [59].

BChE inhibition showed a divergent pattern, with the strongest activity recorded for sample 10 (2.99 mg GALAE/g), extracted at 50 °C with a moderate solvent ratio. This divergence between AChE and BChE inhibition profiles suggests that the two enzyme targets respond differently to the polyphenol composition of the extracts, which is in turn modulated by extraction conditions. In advanced Alzheimer’s disease, AChE activity declines while BChE activity increases, suggesting a compensatory role for BChE in acetylcholine hydrolysis, which underscores the clinical relevance of targeting both enzymes in the evaluation of neuroprotective plant extracts.

Tyrosinase inhibition. Tyrosinase inhibitory activity ranged from 49.63 to 55.03 mg KAE/g, with samples 11 and 12 again performing best. Tyrosinase is a copper-containing monooxygenase responsible for melanin biosynthesis and enzymatic browning, and its inhibition is of relevance in both dermatological and food technology applications. Among polyphenols, quercetin has been identified as a potent tyrosinase inhibitor, with inhibitory activity comparable to that of the reference compound kojic acid. Given the well-documented presence of quercetin in apple pomace [51] and its established tyrosinase inhibitory potential, the superior performance of samples 11 and 12 may reflect their higher overall phenolic content, which is generally associated with greater concentrations of tyrosinase-inhibiting constituents. Previous studies on apple pomace extracts reported tyrosinase inhibitory activity with IC_50_ values between 13.2 and 30.8 µg/mL [60,61,62], confirming that apple pomace represents a relevant source of tyrosinase-inhibiting bioactive compounds regardless of the extraction approach employed. The relatively narrow overall range of tyrosinase inhibition across all samples (approximately 10% variation) suggests that the compounds responsible for this activity are reliably extracted across a wide range of UAE conditions.

α-Amylase and α-glucosidase inhibition. α-Amylase inhibition showed the widest relative variation of all tested enzymes (0.45–0.89 mmol ACAE/g), with sample 15 demonstrating the highest activity. This finding is particularly noteworthy given that sample 15 yielded some of the lowest TP values, indicating that α-amylase inhibitory activity is not directly correlated with total phenolic content but rather reflects the selective enrichment of specific compound classes under certain extraction conditions. Galloylated catechins and procyanidins are among the most potent α-amylase inhibitors among polyphenols, with hydrophobic forces governing their binding to the hydrophobic catalytic centre of the enzyme [60]. The combination of high temperature (75 °C), prolonged extraction time, and high solvent-to-solid ratio employed for sample 15 may have favoured the recovery of constituents contributing to α-amylase inhibition, potentially accounting for the markedly higher activity despite the lower total phenolic content. Quercetin, chlorogenic acid, and phloridzin, all characteristic phenolics of apple, have been associated with reduced postprandial hyperglycaemia primarily through inhibition of carbohydrate-digesting enzymes and intestinal glucose transporters, further supporting the antidiabetic relevance of the enzyme inhibitory activities observed in the present study [60,61]. In contrast, α-glucosidase inhibition showed the narrowest range of all assays (1.01–1.25 mmol ACAE/g), suggesting that the compounds responsible for this activity are broadly and consistently extracted across the full range of UAE conditions tested. Glycosylated forms of flavonoids have been reported to be weaker α-glucosidase inhibitors than their aglycone counterparts, and since aqueous extraction systems are known to preferentially recover glycosylated flavonoid forms [60,63], this may explain the relatively uniform and moderate α-glucosidase inhibitory activity observed across samples. The notably lower activity of sample 12 (1.01 mmol ACAE/g) may reflect differences in polyphenol composition under high-dilution, low-temperature conditions compared to other extraction regimes, as changes in extraction parameters are known to differentially affect the recovery of glycosylated versus aglycone flavonoid forms [48,63].

Generally, these results demonstrate that UAE conditions exert enzyme-specific effects on the inhibitory bioactivity profile of apple pomace extracts. Conditions favouring higher antioxidant capacity and cholinesterase/tyrosinase inhibition (samples 11 and 12) do not simultaneously yield the highest α-amylase inhibitory activity, for which more stringent extraction conditions (sample 15) appear advantageous. These findings highlight the importance of defining the intended biological target before extraction optimisation and underscore the potential of apple pomace as a multi-target source of bioactive compounds with relevance to neurodegenerative, metabolic, and pigmentation-related pathological conditions.

### 3.5. Colour Correlation Analysis

**Figure 2:**
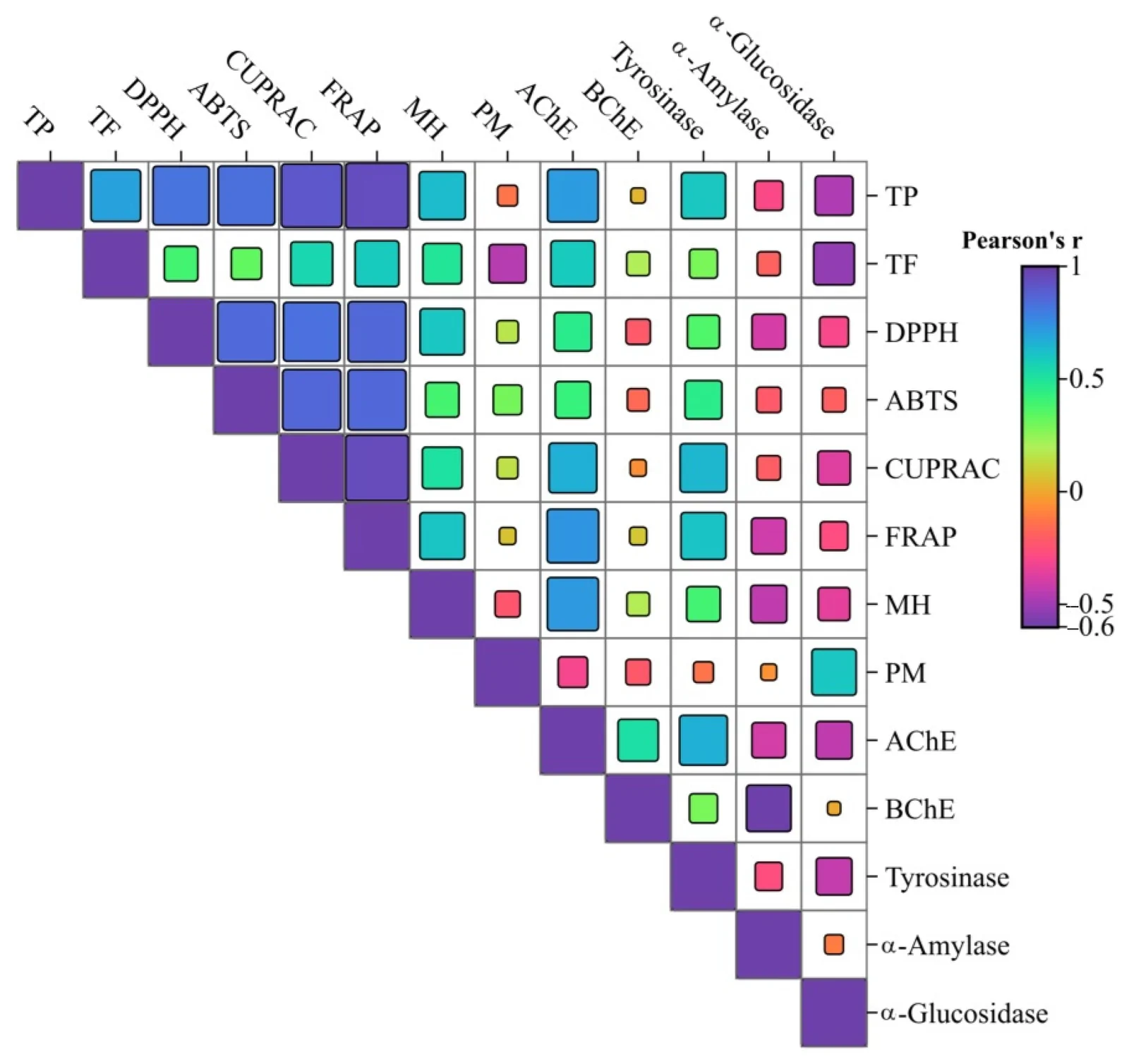
The correlation analysis between observed responses.

The correlation analysis performed across all apple pomace extract samples revealed strong positive relationships among TP, TF, and antioxidant assays (DPPH, ABTS, CUPRAC, and FRAP), with all correlation coefficients exceeding 0.706 and significance at *p* < 0.001 (Figure 2).

A strong interdependence between the chemical composition and bioactivity of the apple pomace UAE extracts was confirmed through correlation analysis. Total phenolic content (TP) exhibited very strong positive correlations with all antioxidant assays, including DPPH, ABTS, CUPRAC, and FRAP (r = 0.827–0.945, *p* < 0.001), demonstrating that phenolic constituents are the main contributors to the radical-scavenging and reducing capacities of the extracts. Total flavonoids (TF) also correlated with antioxidant activity, although to a lesser extent (r = 0.555–0.592), indicating that other phenolic classes beyond flavonoids play a substantial role in antioxidant performance. Antioxidant assays themselves were tightly intercorrelated (r > 0.85), confirming that the extracts show consistent behaviour across different oxidative mechanisms. TP also displayed significant positive associations with acetylcholinesterase (AChE) and tyrosinase inhibition (r = 0.598–0.718), while FRAP and CUPRAC showed the strongest associations with AChE (r = 0.735 and 0.664, respectively), suggesting that reducing-type antioxidants contribute to neuroprotective and anti-browning activities. In contrast, digestive enzyme inhibition displayed weak or non-significant relationships with phenolic content; only α-glucosidase inhibition showed a significant negative correlation with TF (r = −0.540, *p* < 0.05), implying that compounds other than flavonoids drive this activity. Overall, these results highlight phenolic-rich fractions as key determinants of antioxidant potential and cholinesterase/tyrosinase inhibitory effects in apple pomace extracts, whereas carbohydrate-hydrolysing enzyme inhibition appears to be governed by distinct chemical components.

### 3.6. Principal Component Analysis (PCA)

**Figure 3:**
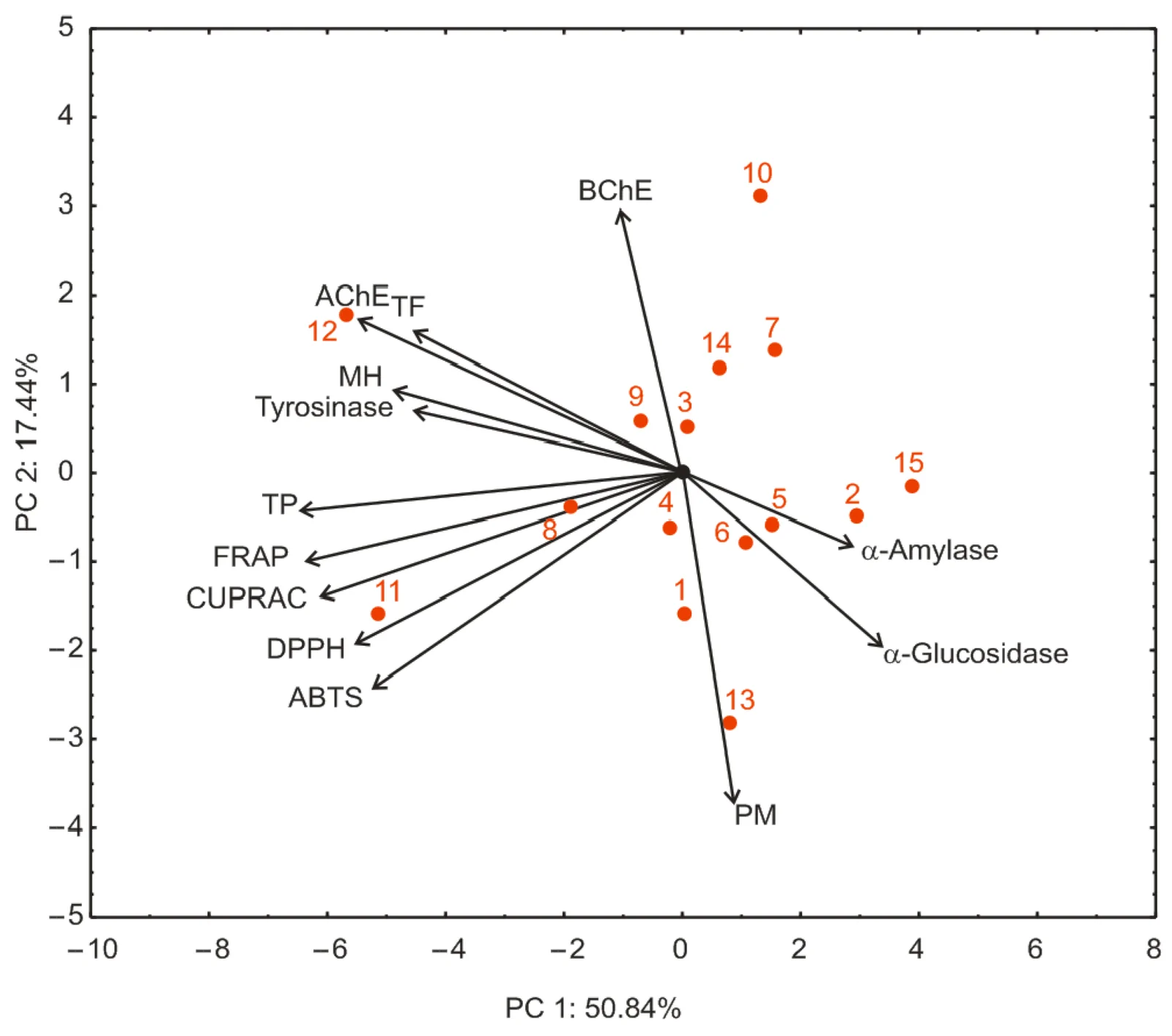
Principal component analysis (PCA) of apple pomace extracts samples based on observed responses.

The PCA biplot revealed clear differentiation among apple pomace extracts based on their phenolic composition, antioxidant potential, and enzyme-inhibitory activities (Figure 3). The first two principal components explained 68.28% of the total variance (PC1: 50.84%, PC2: 17.44%). Samples 11 and 12 were positioned strongly in the negative PC1 direction, closely aligned with TP, TF, DPPH, ABTS, FRAP, and CUPRAC vectors, confirming that these extracts possessed the highest levels of antioxidant-related constituents. Sample 12 also aligned with AChE and MH activities, indicating richer extraction of compounds with cholinesterase-inhibitory and metal-chelating effects. In contrast, samples 2, 5, and 15 were positioned in the positive PC1 direction, correlating strongly with α-amylase and α-glucosidase inhibitory activities, reflecting the extraction of compounds exerting pronounced antidiabetic effects under higher-temperature or extended extraction conditions. Sample 10 showed a strong positive PC2 association with BChE inhibition, suggesting a unique composition distinct from other extracts. Sample 13 grouped separately along the negative PC2 axis, driven by its high contribution to the PM assay. Overall, PCA clearly separated extracts into antioxidant-rich samples (11, 12), enzyme-inhibition-dominant samples (2, 5, 15), and those with distinctive selective bioactivities (10, 13), demonstrating that extraction parameters significantly shaped the chemical and bioactive profiles of apple pomace.

### 3.7. Hierarchical Cluster Analysis (HCA)

**Figure 4:**
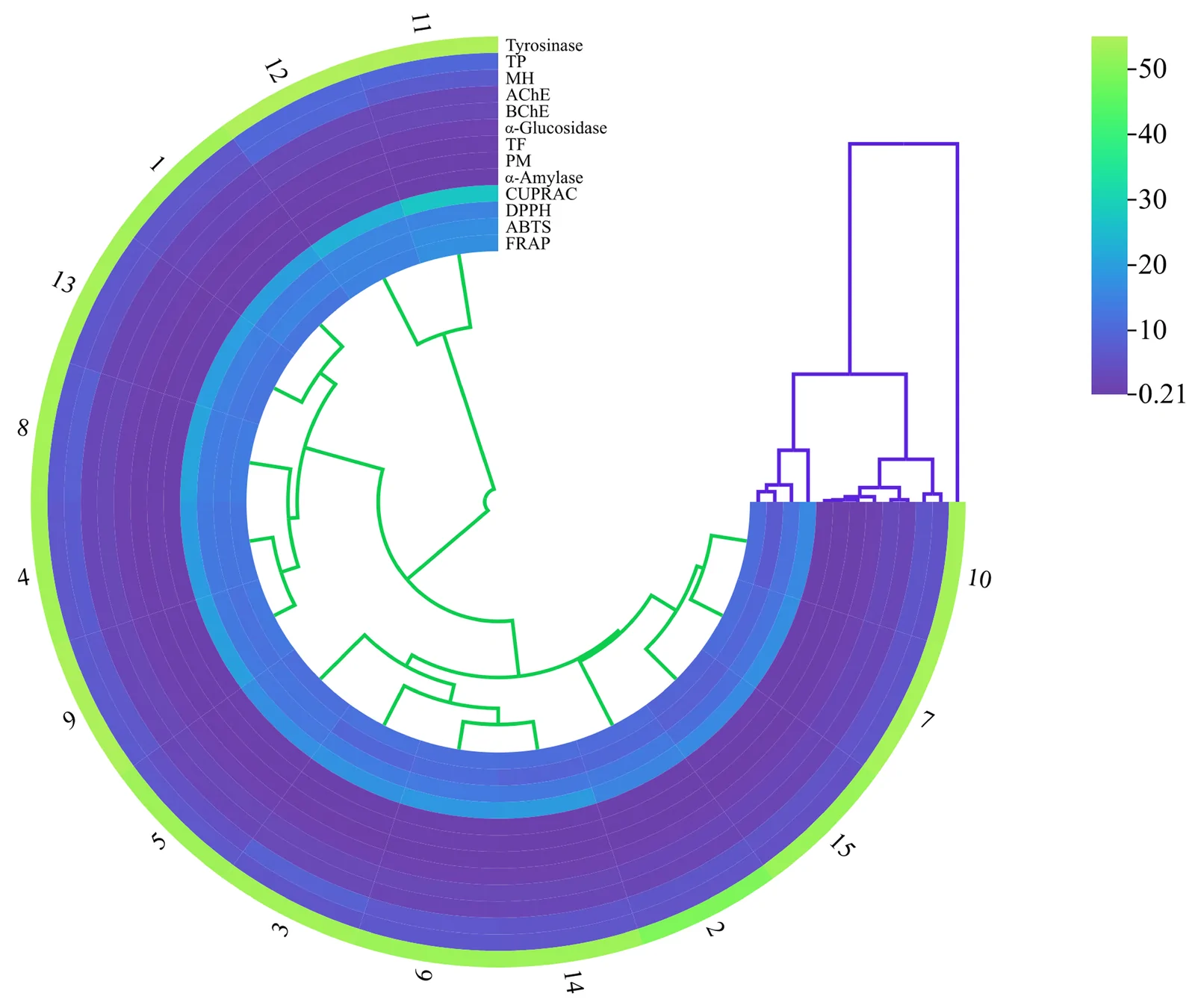
Hierarchical cluster analysis of apple pomace extract samples based on observed responses.

The hierarchical cluster analysis (complete linkage, Manhattan distances) combined with the circular heatmap provides an integrated overview of the relationships between ultrasound-assisted extraction (UAE) conditions (samples 1–15) and the measured bioactive responses, including total phenolics (TPs), total flavonoids (TFs), antioxidant assays (DPPH, ABTS, CUPRAC, FRAP, MH, and PM), and enzyme inhibitory activities (AChE, BChE, tyrosinase, α-amylase, and α-glucosidase; Figure 4). The outer circular heatmap illustrates the intensity of each variable across all samples, where the colour gradient (purple to green) reflects increasing activity levels. Samples 11 and 12 clearly stand out with the highest intensity (green shades) across most parameters, confirming their higher extraction efficiency. In contrast, samples such as 1, 3, and 15 are dominated by lower-intensity colours (purple-blue), indicating less efficient extraction conditions.

The inner dendrogram reveals the clustering pattern of samples based on similarity in their biochemical profiles. Two main clusters can be observed: one grouping high-performing samples (notably 11 and 12, along with several moderately active extracts such as 8 and 9), and another comprising samples with lower or moderate activities. This clustering indicates that extraction parameters strongly influence the overall bioactivity profile, with differences in temperature and solvent-to-solid ratio appearing to contribute more strongly to the observed clustering patterns, while extraction time showed a less pronounced association.

Additionally, the clustering of variables (right-side dendrogram) suggests strong associations among antioxidant assays (DPPH, ABTS, CUPRAC, and FRAP) and phenolic-related measures (TP and TF), confirming that phenolic compounds are the primary contributors to antioxidant capacity. Enzyme inhibition parameters, particularly AChE, BChE, and tyrosinase, are closely grouped with these antioxidant metrics, indicating shared underlying bioactive compounds. In contrast, α-amylase and α-glucosidase show slightly different clustering behaviour, suggesting a more selective extraction of compounds responsible for carbohydrate-hydrolysing enzyme inhibition.

### 3.8. Hyperparameters Results

The hyperparameter optimization results for the ANN, RF, and SVM models reveal several consistent statistical patterns that reflect both the structure of the dataset and the complexity of relationships between predictors and the biochemical responses.

For the ANN models, the optimal architectures indicate that the predictive relationships are generally nonlinear but do not require large networks [64]. The optimal number of neurons in the hidden layer ranges from one to four across all target variables, with decay values of either 0.01 or 0.1. Targets such as TF, FRAP, and tyrosinase consistently favour more complex architectures, typically involving three to five neurons and lower decay values (0.01), suggesting that these biochemical responses are governed by more intricate nonlinear dependencies that benefit from reduced regularization. In contrast, parameters such as TP, CUPRAC, PM, and several enzyme activities show optimal performance with smaller architectures (one to three neurons) and moderate decay, indicating that comparatively simpler relationships dominate their predictive structure. The ANN patterns therefore suggest variability in response complexity across biochemical targets, with some traits being adequately explained by basic nonlinear functions, while others require more modelling capacity.

The random forest models exhibit a markedly different pattern, with the optimal mtry parameter, representing the number of variables considered at each split, most frequently equal to 1. This dominance of low mtry values indicates substantial redundancy among predictors and suggests that random forest performance improves when the trees are built using highly randomized, minimally correlated subsets of predictors. Only a small number of responses (ABTS, PM, and AChE) require slightly higher mtry values (2–3), implying that these specific outputs involve more distributed information across predictors and thus benefit from considering a broader feature set at each node. The prevalence of mtry = 1 across most biochemical parameters confirms that the dataset features correlated predictors and limited benefit from exhaustive feature consideration during tree construction.

The SVM model results are exceptionally stable [28], with all target variables selecting the same combination of sigma = 0.1 and cost parameter C = 1. This repeated pattern indicates that a moderately smooth RBF kernel and balanced margin complexity are suitable for modelling all biochemical and enzymatic responses. The uniform behaviour across targets suggests that the predictor–response relationships are of comparable nonlinearity and that the SVM model is insensitive to minor changes in output structure. This stability suggests that a common tuning configuration was suitable for the investigated dataset, although the limited number of independent experimental observations precludes conclusions regarding broader model generalizability.

Taken together, the hyperparameter results demonstrate that the biochemical and enzymatic traits share underlying structural similarities [65] but differ in the degree of nonlinearity and predictor interactions required for optimal prediction. ANN models capture this variability through changes in hidden-layer size and regularization strength [56], RF models highlight strong predictor redundancy [66], and SVM models exhibit relatively consistent tuning behaviour across the investigated targets [28]. This combination of model characteristics supports the conclusion that the dataset is moderately nonlinear, predictor variables are intercorrelated, and response surfaces differ in complexity but remain structurally consistent enough to support stable SVM configuration and highly randomized RF trees.

### 3.9. Verification of Models

The predictive performance of the ANN, RF, SVM, and hybrid models evaluated using leave-one-condition-out cross-validation (LOOCV) is summarized in Table 5.

Model performance was strongly response-dependent, with substantial differences observed between training and held-out test predictions. For TP, the ANN and hybrid models showed the highest test R^2^ values (0.698 and 0.689, respectively), whereas for TF the hybrid model provided the highest test R^2^ (0.618). For DPPH, RF and SVM showed comparable predictive performance (test R^2^ = 0.607 and 0.589, respectively), while the ANN model yielded a negative test R^2^ (−0.360), indicating poor predictive performance for held-out conditions for this response. Among the antioxidant responses, the strongest predictive performance was observed for ABTS and FRAP. The hybrid model achieved the highest test R^2^ for ABTS (0.920), accompanied by relatively low test RMSE (0.506) and SSE (3.070), whereas ANN provided the highest test R^2^ for FRAP (0.848; RMSE = 0.514; SSE = 3.167). RF showed the best predictive performance for CUPRAC (test R^2^ = 0.698) and MH (test R^2^ = 0.474), although the latter remained moderate. Prediction of PM was comparatively weak, with test R^2^ values ranging from −0.031 for SVM to 0.488 for the hybrid model.

Prediction of enzyme-inhibitory responses was more challenging and varied considerably among endpoints. For α-amylase, ANN showed the strongest predictive performance (test R^2^ = 0.868), followed by RF (0.747) and the hybrid model (0.725). Tyrosinase prediction was moderate, with RF providing the highest test R^2^ (0.403). In contrast, AChE, BChE, and particularly α-glucosidase showed limited predictive performance. The highest test R^2^ values for AChE and BChE were only 0.258 for RF and 0.166 for ANN, respectively, while all models produced negative test R^2^ values for α-glucosidase. Negative R^2^ values indicate that, for these responses, prediction of held-out conditions was poorer than prediction based on the response mean and should therefore not be interpreted as evidence of useful generalization.

Overall, no single modelling approach consistently outperformed the others across all biochemical responses. RF generally provided comparatively stable predictive performance for several endpoints, whereas the hybrid model performed particularly well for ABTS and TF and provided competitive predictions for several other responses. ANN achieved strong performance for selected responses, including FRAP and α-amylase, but showed marked instability for others, such as DPPH and AChE. These findings demonstrate the response-dependent nature of model performance and emphasize the importance of evaluating predictive accuracy separately for each biochemical endpoint rather than inferring general model superiority from training performance alone. Given the limited number of independent experimental conditions (n = 15), the LOOCV results should be regarded as exploratory estimates of predictive performance within the investigated experimental domain.

Future studies employing larger experimental datasets would be expected to improve the predictive accuracy, stability, and generalizability of the developed models [67]. Many of the observed limitations, particularly the instability between training and test R^2^ values, inflated test RMSE and SSE for certain biochemical parameters, and evident overfitting in the ANN models, are characteristic of modelling on relatively small datasets [35]. With limited sample diversity, models are unable to capture the full variability and underlying structure of the biochemical responses [68], leading to high variance in predictions and reduced robustness when applied to unseen data. In particular, ANN and SVM models, which typically require larger datasets to optimize nonlinear decision boundaries, would benefit from additional data by exhibiting improved convergence and reduced overfitting. Random forest and hybrid models would also gain from broader sampling through enhanced tree diversity and more stable ensemble aggregation.

Therefore, the presented models should be interpreted as proof-of-concept predictive tools rather than fully generalizable models. Future studies with expanded datasets and independent external validation are required to further assess model robustness and predictive reliability.

### 3.10. Standard Score Optimization

All 13 biochemical responses were considered desirable at higher values and were therefore evaluated using a larger-the-better standardized-score criterion. The analysis identified sample 11 (30 min, 75 °C, 10 mL/g solvent-to-solid ratio) as the condition with the highest overall biochemical performance score (0.818), followed by sample 12 (10 min, 25 °C, 20 mL/g; 0.686) and sample 8 (30 min, 25 °C, 20 mL/g; 0.603). The superior overall score of sample 11 resulted from its consistently high phenolic, flavonoid, antioxidant, and selected enzyme-inhibitory responses. In contrast, sample 12 combined the second-highest overall biochemical score with substantially milder extraction conditions, particularly in terms of extraction time and temperature. Thus, although sample 11 showed the highest integrated biochemical performance, sample 12 represented a particularly favourable experimental condition when biochemical performance and process severity were considered together. This distinction was subsequently addressed through multi-objective NSGA-II optimization, in which biochemical performance was balanced against process intensity.

### 3.11. Optimization of Parameters

The optimization of extraction process parameters was performed using a multi-objective evolutionary approach based on the NSGA-II algorithm [69], allowing the simultaneous consideration of biochemical performance and process efficiency. Three experimentally adjustable factors—extraction time, temperature, and solvent-to-solid ratio—were defined as decision variables, with their feasible ranges constrained by the minimum and maximum values investigated experimentally [70]. To favour less resource-intensive extraction conditions, extraction time, temperature, and solvent-to-solid ratio were included as minimization objectives, whereas all biochemical responses were treated as maximization objectives.

All measured biochemical responses, including TP, TF, DPPH, ABTS, CUPRAC, FRAP, MH, PM, AChE, BChE, tyrosinase, α-amylase, and α-glucosidase, were treated as maximization objectives, consistent with the larger-the-better criterion applied in the standardized-score analysis. For each candidate solution generated by the algorithm, response values were estimated using the hybrid predictive model obtained by averaging the outputs of ANN, random forest, and SVM models [71]. The hybrid model was used as a common surrogate across all responses to maintain a consistent modelling framework during multi-objective optimization rather than because it exhibited the highest predictive performance for every individual response. Equal weighting of the ANN, RF, and SVM predictions was adopted as a neutral ensemble strategy that avoids introducing additional model-specific weighting parameters that could not be robustly estimated from the limited number of independent experimental conditions. As demonstrated by the LOOCV results in Table 5, the relative performance of the individual models was response-dependent; therefore, the hybrid model should be interpreted as a pragmatic ensemble surrogate for Pareto exploration rather than as a universally superior predictive model. NSGA-II was executed using a population of 100 solutions over 50 evolutionary generations, generating a Pareto front representing non-dominated trade-offs between biochemical performance and process severity [72].

To select a single representative solution from the Pareto set, all objective values were first normalized to a common dimensionless scale. The representative compromise was then selected using a minimum-distance-to-ideal criterion, i.e., the Pareto solution with the smallest Euclidean distance from the ideal objective vector, in which biochemical responses were maximized and process-intensity variables were minimized. This criterion was used solely to select a representative compromise from the non-dominated solution set and does not imply that the selected point represents a unique global optimum.

**Figure 5:**
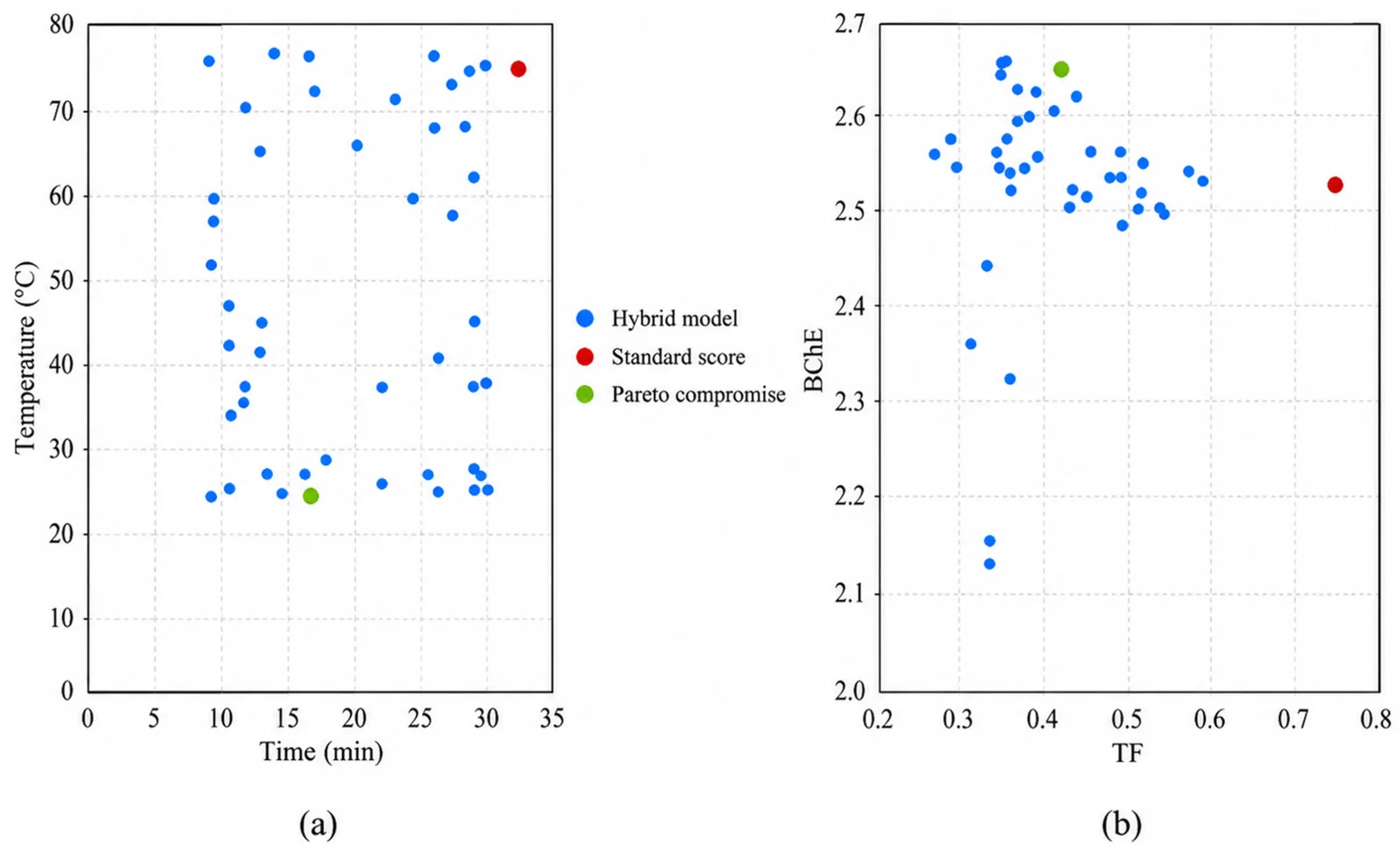
Visualization of the multi-objective optimization of ultrasound-assisted extraction conditions for apple pomace. (**a**) Distribution of candidate solutions across extraction time and temperature, with the solvent-to-solid ratio represented by point size; (**b**) relationship between predicted total flavonoid content (TF) and butyrylcholinesterase (BChE) inhibitory activity for the candidate solutions. Blue points represent candidate solutions generated using the hybrid model, the red point represents the experimentally identified condition with the highest standardized biochemical performance, and the green point represents the selected model-derived Pareto compromise, corresponding to approximately 15.1 min, 25.7 °C, and 13.7 mL/g.

The multi-objective optimization identified a representative compromise solution at approximately 15.1 min, 25.7 °C, and a solvent-to-solid ratio of 13.7 mL/g, Figure 5. This solution represents a non-dominated point on the Pareto front, indicating that improvement in one or more objectives would require deterioration in at least one other objective. Importantly, the selected model-derived Pareto compromise was characterized by a relatively low extraction temperature and moderate extraction time and solvent requirement, demonstrating that high biochemical performance can be achieved without applying the most severe processing conditions.

The resulting Pareto-optimal parameter combinations provide a data-driven basis for selecting extraction conditions that balance desirable phytochemical and enzyme-inhibitory responses with processing requirements [73].

Comparison of the Pareto-optimal solutions with the experimentally evaluated conditions further showed that sample 12 (10 min, 25 °C, 20 mL/g) remained a Pareto-competitive experimental condition. Although sample 11 exhibited the highest overall standardized biochemical score, its substantially longer extraction time and higher temperature (30 min and 75 °C) increased process severity. In contrast, sample 12 provided a favourable balance between high biochemical performance and mild processing conditions. Thus, the standardized-score analysis and NSGA-II optimization provide complementary information: the former identifies the experimental condition with the strongest integrated biochemical performance, whereas the latter identifies non-dominated compromises between biochemical responses and processing requirements.

### 3.12. Experimental Verification and Relation to the Pareto-Optimal Solution

An independent experimental verification was performed at the conditions corresponding to sample 12 (10 min, 25 °C, and 20 mL/g), which had shown high overall biochemical performance in the original experimental dataset. The verification experiment was independently prepared and analysed using the same experimental procedure as the original extraction. The resulting biochemical responses were compared with the original experimental measurements obtained at the same conditions to assess experimental reproducibility.

The NSGA-II analysis identified a representative Pareto compromise at approximately 15.1 min, 25.7 °C, and 13.7 mL/g. This computationally selected compromise differs from the experimentally verified sample 12 condition and therefore should not be interpreted as having been independently experimentally validated. Nevertheless, sample 12 remained non-dominated relative to the returned Pareto solutions and therefore represents a Pareto-competitive experimental condition. Importantly, the two approaches address complementary aspects of process selection: sample 12 demonstrates experimentally verified high biochemical performance under mild extraction conditions, whereas the NSGA-II compromise represents a model-based balance among biochemical responses, extraction time, temperature, and solvent requirement. Further experimental validation of the exact Pareto-compromise conditions is warranted in future studies.

The independent experimental verification was performed at the conditions corresponding to sample 12 (10 min, 25 °C, and 20 mL/g). The NSGA-II compromise (15.1 min, 25.7 °C, and 13.7 mL/g) represents a model-derived Pareto-optimal solution and was not independently experimentally validated. Experimental difference (%) was calculated between the original sample 12 measurement and its independently repeated experimental measurement. Model-prediction errors for the training and held-out test observations generated within the LOOCV procedure are reported in Table 5 using R^2^, RMSE, SSE, and AARD; percentage prediction differences were not calculated in Table 6 because the biochemical responses are expressed in different units and span markedly different numerical ranges. The response-dependent deviations between the experimental values and hybrid-model predictions, particularly for TP and TF, further emphasize the exploratory nature of the predictive models developed from the limited experimental dataset.

An important limitation of the present extraction strategy is that water was used as the sole solvent, and solvent composition was not included among the investigated optimization variables. Apple pomace contains chemically diverse phenolic constituents whose polarity and aqueous solubility differ substantially; consequently, a single aqueous solvent system cannot be expected to provide equally efficient recovery of all phenolic subclasses. The comparatively lower TF values observed in the present study should therefore be interpreted within the context of the selected aqueous extraction system rather than as evidence of globally optimal flavonoid recovery. Accordingly, the conditions identified by the present modelling and multi-objective optimization represent favourable conditions within the investigated aqueous UAE domain. Future studies should expand the experimental space to include solvent composition as an additional factor and directly compare water with food-compatible aqueous-organic or other green solvent systems.

## 4. Conclusions

The results of this study demonstrate the potential of integrating ultrasound-assisted extraction with machine learning-based modelling and multi-objective optimization for the recovery of bioactive compounds from apple pomace. Extraction conditions influenced phenolic and flavonoid contents, antioxidant capacity, and enzyme-inhibitory activities, with distinct response patterns observed across the investigated conditions. Among the experimentally tested conditions, sample 11 exhibited the highest overall standardized biochemical performance, whereas sample 12 (10 min, 25 °C, and 20 mL/g) combined high biochemical performance with substantially milder extraction conditions.

ANN, RF, SVM, and a hybrid ensemble approach were used to model the relationships between extraction parameters and biochemical responses. Although the hybrid model provided a balanced predictive performance, the relatively small number of unique experimental conditions limits the generalizability of the developed models. Accordingly, the modelling results should be interpreted as an exploratory data-driven framework, and future studies should include larger experimental datasets, external validation, and compound-level characterization.

With all desirable biochemical responses, including enzyme-inhibitory activities, treated as maximization objectives, NSGA-II identified a representative Pareto compromise at approximately 15.1 min, 25.7 °C, and 13.7 mL/g. This solution represents a model-derived trade-off between biochemical performance and processing requirements. Importantly, the exact Pareto-compromise condition was not independently experimentally validated. Instead, independent experimental verification was performed for sample 12 and demonstrated good reproducibility of the experimentally measured biochemical responses. Sample 12 also remained non-dominated relative to the returned Pareto solutions, supporting its relevance as a Pareto-competitive experimental condition under mild processing conditions.

The identified conditions should therefore be regarded as a Pareto-based compromise within the investigated aqueous UAE domain rather than as universally optimal conditions for the exhaustive recovery of all phenolic subclasses. In particular, the comparatively lower flavonoid recovery reflects the deliberate use of water as the sole extraction solvent and further emphasizes that the identified conditions are specific to the investigated aqueous extraction domain. Optimization of solvent composition was beyond the scope of the present study and represents an important direction for future research. The findings demonstrate the value of combining multi-response biochemical screening with machine learning-assisted multi-objective optimization for identifying promising extraction regimes. Further experimental validation of the model-derived Pareto compromise and expansion of the experimental dataset are required before the selected model-derived conditions can be considered robust or broadly generalizable.

## Figures and Tables

**Table 1:** Experimental design for ultrasound-assisted extraction of apple pomace.

Sample	Time [min]	Temperature [°C]	Solvent-to-Solid Ratio [mL/g]
1	10	25	10
2	10	50	15
3	10	75	20
4	20	25	15
5	20	50	10
6	20	50	20
7	20	75	15
8	30	25	20
9	30	50	10
10	30	50	15
11	30	75	10
12	10	25	20
13	20	25	10
14	10	75	15
15	30	75	20

**Table 2:** Total phenolic and flavonoid contents ^1^ in the tested extracts.

Apple Pomace	TP (mg GAE/g) ^2^	TF (mg RE/g) ^3^
1	6.43 ± 0.14 ^cde^	0.24 ± 0.02 ^a^
2	5.52 ± 0.04 ^ab^	0.47 ± 0.06 ^c^
3	5.99 ± 0.04 ^bc^	0.24 ± 0.01 ^a^
4	6.45 ± 0.05 ^cde^	0.41 ± 0.06 ^c^
5	6.45 ± 0.04 ^cde^	0.39 ± 0.05 ^bc^
6	6.28 ± 0.30 ^bcd^	0.46 ± 0.05 ^c^
7	5.82 ± 0.20 ^bc^	0.43 ± 0.03 ^c^
8	7.27 ± 0.30 ^e^	0.63 ± 0.02 ^d^
9	6.87 ± 0.11 ^de^	0.45 ± 0.04 ^c^
10	5.46 ± 0.03 ^ab^	0.47 ± 0.04 ^c^
11	9.97 ± 0.23 ^f^	0.72 ± 0.04 ^de^
12	9.92 ± 0.92 ^f^	0.79 ± 0.03 ^e^
13	6.30 ± 0.07 ^bcd^	0.21 ± 0.01 ^a^
14	5.88 ± 0.10 ^bc^	0.62 ± 0.02 ^d^
15	4.70 ± 0.09 ^a^	0.29 ± 0.02 ^ab^

^1^ Values are means ± SD of three measurements. The means within each column with different letters (a–f) differ significantly (*p* < 0.05); ^2^ mg Gallic acid equivalents per gram of dry apple pomace; ^3^ mg rutin equivalents per gram of dry apple pomace.

**Table 3:** Antioxidant properties ^1^ of the tested extracts.

Apple Pomace	DPPH (mg TE/g) ^2^	ABTS (mg TE/g) ^2^	CUPRAC (mg TE/g) ^2^	FRAP (mg TE/g) ^2^	MH (mg EDTAE/g) ^3^	PM (mmol TE/g) ^4^
1	13.99 ± 0.49 ^def^	15.01 ± 0.07 ^h^	20.14 ± 0.33 ^gh^	12.39 ± 0.43 ^de^	5.58 ± 0.35 ^ab^	0.80 ± 0.03 ^g^
2	13.18 ± 0.29 ^cde^	9.71 ± 0.15 ^bc^	15.42 ± 0.11 ^a^	10.59 ± 0.11 ^b^	5.71 ± 0.61 ^ab^	0.57 ± 0.01 ^bcd^
3	13.84 ± 0.25 ^def^	9.59 ± 0.22 ^bc^	17.97 ± 0.28 ^cd^	11.90 ± 0.09 ^cd^	8.41 ± 0.86 ^de^	0.63 ± 0.03 ^cde^
4	13.26 ± 0.30 ^cde^	12.41 ± 0.66 ^efg^	19.23 ± 0.42 ^efg^	12.47 ± 0.36 ^de^	7.22 ± 0.58 ^cd^	0.81 ± 0.03 ^g^
5	12.83 ± 0.30 ^bcd^	12.18 ± 0.27 ^ef^	17.04 ± 0.42 ^bc^	11.20 ± 0.34 ^bc^	4.96 ± 0.60 ^a^	0.64 ± 0.07 ^cde^
6	13.37 ± 0.83 ^def^	10.70 ± 0.54 ^cd^	18.50 ± 0.23 ^de^	11.23 ± 0.52 ^bc^	6.56 ± 0.16 ^bc^	0.76 ± 0.04 ^fg^
7	12.00 ± 0.42 ^abc^	9.41 ± 0.15 ^b^	17.05 ± 0.34 ^bc^	10.91 ± 0.24 ^b^	5.31 ± 0.34 ^ab^	0.49 ± 0.01 ^b^
8	14.26 ± 0.38 ^efg^	13.47 ± 0.58 ^g^	20.87 ± 0.24 ^h^	13.75 ± 0.35 ^f^	8.22 ± 0.35 ^d^	0.67 ± 0.04 ^def^
9	13.28 ± 0.42 ^cde^	11.82 ± 0.17 ^de^	19.96 ± 0.28 ^fgh^	12.94 ± 0.16 ^ef^	6.44 ± 0.40 ^bc^	0.55 ± 0.01 ^bc^
10	11.63 ± 0.33 ^ab^	7.28 ± 0.53 ^a^	15.89 ± 0.72 ^a^	10.71 ± 0.25 ^b^	6.05 ± 0.11 ^abc^	0.48 ± 0.01 ^b^
11	15.44 ± 0.59 ^g^	16.84 ± 0.28 ^i^	27.02 ± 0.73 ^j^	17.12 ± 0.28 ^h^	7.12 ± 0.33 ^cd^	0.72 ± 0.04 ^efg^
12	15.51 ± 0.64 ^g^	15.31 ± 0.47 ^h^	22.96 ± 0.28 ^i^	14.91 ± 0.11 ^g^	9.59 ± 0.55 ^e^	0.33 ± 0.02 ^a^
13	14.71 ± 0.29 ^fg^	13.10 ± 0.51 ^fg^	19.89 ± 0.06 ^fgh^	12.18 ± 0.26 ^de^	5.57 ± 0.09 ^ab^	0.75 ± 0.05 ^fg^
14	13.04 ± 0.46 ^cde^	9.07 ± 0.10 ^b^	19.01 ± 0.17 ^def^	10.98 ± 0.15 ^b^	5.67 ± 0.29 ^ab^	0.56 ± 0.05 ^bc^
15	11.17 ± 0.38 ^a^	8.53 ± 0.45 ^b^	16.10 ± 0.31 ^ab^	8.99 ± 0.05 ^a^	5.59 ± 0.29 ^ab^	0.60 ± 0.03 ^cd^

^1^ Values are means ± SD of three measurements. The means within each column with different letters (a–j) differ significantly (*p* < 0.05); ^2^ mg Trolox equivalents per g of dry apple pomace, ^3^ mg EDTAE per g of dry apple pomace; ^4^ mmol Trolox equivalents per g of dry apple pomace.

**Table 4:** Enzyme inhibitor activity ^1^ of apple pomace extracts under different extraction conditions.

Apple Pomace	AChE (mg GALAE/g) ^2^	BChE (mg GALAE/g) ^2^	Tyrosinase (mg KAE/g) ^3^	α-Amylase (mmol ACAE/g) ^4^	α-Glucosidase (mmol ACAE/g) ^4^
1	2.30 ± 0.05 ^cd^	2.69 ± 0.20 ^d^	53.28 ± 0.43 ^bc^	0.55 ± 0.02 ^cde^	1.20 ± 0.01 ^bc^
2	2.11 ± 0.05 ^a^	2.39 ± 0.02 ^bc^	49.63 ± 0.95 ^a^	0.59 ± 0.01 ^ef^	1.25 ± 0.01 ^c^
3	2.46 ± 0.03 ^ef^	2.68 ± 0.06 ^d^	53.38 ± 0.22 ^bc^	0.47 ± 0.01 ^ab^	1.23 ± 0.01 ^c^
4	2.38 ± 0.05 ^de^	2.70 ± 0.08 ^d^	53.65 ± 0.60 ^bcd^	0.49 ± 0.01 ^abc^	1.24 ± 0.01 ^c^
5	2.18 ± 0.03 ^abc^	2.54 ± 0.13 ^cd^	53.14 ± 0.34 ^bc^	0.57 ± 0.01 ^def^	1.23 ± 0.01 ^c^
6	2.14 ± 0.02 ^a^	2.56 ± 0.08 ^cd^	52.79 ± 0.25 ^b^	0.53 ± 0.01 ^bcde^	1.22 ± 0.01 ^c^
7	2.29 ± 0.01 ^bcd^	2.72 ± 0.08 ^de^	54.10 ± 0.66 ^bcd^	0.59 ± 0.01 ^ef^	1.22 ± 0.01 ^c^
8	2.37 ± 0.04 ^de^	2.58 ± 0.04 ^cd^	53.75 ± 0.36 ^bcd^	0.45 ± 0.02 ^a^	1.23 ± 0.01 ^c^
9	2.39 ± 0.01 ^de^	2.68 ± 0.02 ^d^	54.62 ± 0.13 ^cd^	0.52 ± 0.01 ^bcd^	1.20 ± 0.02 ^bc^
10	2.37 ± 0.05 ^de^	2.99 ± 0.09 ^e^	53.98 ± 0.17 ^bcd^	0.45 ± 0.02 ^a^	1.16 ± 0.05 ^bc^
11	2.58 ± 0.01 ^f^	2.53 ± 0.11 ^bcd^	55.02 ± 0.43 ^d^	0.58 ± 0.07 ^def^	1.18 ± 0.01 ^bc^
12	2.58 ± 0.02 ^f^	2.57 ± 0.07 ^cd^	55.03 ± 0.18 ^d^	0.53 ± 0.01 ^bcde^	1.01 ± 0.08 ^a^
13	2.08 ± 0.10 ^a^	2.09 ± 0.13 ^a^	53.99 ± 0.28 ^bcd^	0.58 ± 0.01 ^def^	1.18 ± 0.03 ^bc^
14	2.32 ± 0.04 ^d^	2.56 ± 0.02 ^cd^	53.02 ± 1.18 ^bc^	0.62 ± 0.01 ^f^	1.10 ± 0.07 ^ab^
15	2.16 ± 0.05 ^ab^	2.26 ± 0.06 ^ab^	52.64 ± 0.59 ^b^	0.89 ± 0.01 ^g^	1.18 ± 0.04 ^bc^

^1^ Values are means ± SD of three measurements, means within each column with different letters (a–g) differ significantly (*p* < 0.05); ^2^ mg galantamine equivalents per g of dry apple pomace; ^3^ mg kojic acid per g of dry apple pomace; ^4^ mmol acarbose equivalents per g of dry apple pomace.

**Table 5:** Performance metrics of the developed machine learning models evaluated using leave-one-condition-out cross-validation (LOOCV).

		R^2^		RMSE		SSE		AARD	
Target	Model	Train	Test	Train	Test	Train	Test	Train	Test
TP	ANN	0.956	0.698	0.338	0.354	3.775	1.501	3.865	5.186
	RF	0.902	0.479	0.507	0.464	8.476	2.588	4.915	6.746
	SVM	0.627	0.587	0.989	0.413	32.270	2.050	7.125	5.427
	Hybrid	0.891	0.689	0.535	0.359	9.445	1.545	4.486	5.106
TF	ANN	0.793	0.244	0.086	0.092	0.246	0.101	16.105	15.775
	RF	0.893	0.520	0.062	0.073	0.127	0.064	13.804	14.875
	SVM	0.727	0.572	0.099	0.069	0.324	0.057	18.692	10.844
	Hybrid	0.845	0.618	0.075	0.065	0.183	0.051	15.592	9.829
DPPH	ANN	0.482	−0.360	1.015	0.846	33.977	8.593	5.080	5.310
	RF	0.858	0.607	0.531	0.455	9.317	2.483	3.247	2.712
	SVM	0.529	0.589	0.967	0.465	30.851	2.598	4.973	3.059
	Hybrid	0.702	0.461	0.770	0.533	19.560	3.405	4.165	3.358
ABTS	ANN	0.336	0.662	2.400	1.036	190.083	12.883	15.610	8.530
	RF	0.958	0.847	0.606	0.698	12.119	5.842	3.904	4.547
	SVM	0.673	0.776	1.683	0.843	93.507	8.538	9.431	5.432
	Hybrid	0.750	0.920	1.473	0.506	71.580	3.070	9.022	3.697
CUPRAC	ANN	0.959	0.484	0.639	1.357	13.471	22.094	2.169	5.888
	RF	0.924	0.698	0.873	1.038	25.144	12.929	3.601	4.983
	SVM	0.456	0.224	2.336	1.665	180.030	33.263	6.131	6.363
	Hybrid	0.877	0.550	1.112	1.267	40.828	19.263	3.515	5.056
FRAP	ANN	0.969	0.848	0.364	0.514	4.361	3.167	2.278	3.768
	RF	0.923	0.724	0.574	0.692	10.867	5.739	3.394	5.467
	SVM	0.580	0.560	1.340	0.874	59.268	9.165	5.717	6.905
	Hybrid	0.898	0.768	0.660	0.634	14.357	4.828	3.565	4.677
MH	ANN	0.904	−0.088	0.445	1.001	6.525	12.034	4.925	14.335
	RF	0.882	0.474	0.494	0.697	8.052	5.823	5.697	9.779
	SVM	0.829	0.469	0.593	0.700	11.618	5.873	6.180	9.675
	Hybrid	0.898	0.351	0.459	0.773	6.946	7.175	4.966	11.030
PM	ANN	0.881	0.463	0.048	0.075	0.077	0.068	6.256	10.085
	RF	0.845	0.460	0.055	0.076	0.100	0.069	8.176	10.170
	SVM	0.375	−0.031	0.111	0.105	0.404	0.131	16.514	13.036
	Hybrid	0.794	0.488	0.063	0.074	0.133	0.065	9.716	9.178
AChE	ANN	0.270	−0.973	0.137	0.181	0.616	0.394	4.772	7.152
	RF	0.856	0.258	0.061	0.111	0.121	0.148	2.055	4.041
	SVM	0.607	−0.466	0.100	0.156	0.332	0.292	3.172	5.924
	Hybrid	0.656	−0.286	0.094	0.146	0.291	0.257	3.161	5.635
BChE	ANN	0.697	0.166	0.127	0.153	0.535	0.280	3.848	4.775
	RF	0.766	−0.083	0.112	0.174	0.413	0.364	3.344	5.231
	SVM	0.225	−0.348	0.204	0.194	1.368	0.453	5.810	6.263
	Hybrid	0.656	−0.042	0.136	0.171	0.608	0.350	4.042	5.389
Tyrosinase	ANN	0.512	0.330	0.707	1.486	16.512	26.512	1.041	2.091
	RF	0.772	0.403	0.484	1.403	7.723	23.615	0.689	1.974
	SVM	0.470	0.048	0.737	1.772	17.924	37.664	0.942	2.255
	Hybrid	0.642	0.281	0.606	1.540	12.102	28.442	0.848	2.082
α Amylase	ANN	0.806	0.868	0.044	0.040	0.065	0.019	6.887	5.538
	RF	0.756	0.747	0.050	0.055	0.082	0.037	5.513	5.636
	SVM	0.215	0.188	0.089	0.099	0.263	0.118	7.658	5.225
	Hybrid	0.714	0.725	0.054	0.058	0.096	0.040	6.406	4.899
α Glucosidase	ANN	0.798	−2.539	0.033	0.040	0.036	0.019	2.197	2.541
	RF	0.647	−2.858	0.044	0.041	0.063	0.020	2.859	2.757
	SVM	0.045	−0.688	0.072	0.027	0.170	0.009	3.802	1.865
	Hybrid	0.645	−0.691	0.044	0.027	0.063	0.009	2.626	2.054

**Table 6:** Experimental verification of sample 12 and comparison with the hybrid-model prediction and NSGA-II compromise solution.

Variable	Sample 12 Exp.	Hybrid Model at Sample 12	NSGA-II Compromise	Sample 12 Exp. Validation	Exp. Diff. (%)
Time (min)	10	10	15.10	10	-
T (°C)	25	25	25.70	25	-
Solvent-to-solid ratio (mL/g)	20	20	13.75	20	-
TP	9.92	6.310	6.341	9.89	0.30
TF	0.79	0.427	0.387	0.79	0.00
DPPH	15.51	13.061	13.296	15.32	1.23
ABTS	15.31	12.350	12.604	15.15	1.05
CUPRAC	22.96	17.986	19.458	22.95	0.04
FRAP	14.91	12.188	12.164	14.84	0.47
MH	9.59	7.209	6.569	9.47	1.25
PM	0.33	0.750	0.730	0.33	0.00
AChE	2.58	2.234	2.264	2.55	1.16
BChE	2.57	2.624	2.652	2.52	1.95
Tyrosinase	55.03	50.189	53.229	54.32	1.29
α-Amylase	0.53	0.525	0.529	0.52	1.89
α-Glucosidase	1.01	1.227	1.215	1.00	0.99

## Data Availability

The original contributions presented in this study are included in the article, and further inquiries can be directed to the corresponding author.

## Abbreviations

The following abbreviations are used in this manuscript:

AARD	Absolute average relative deviation
ABTS	2.2′-azino-bis (3-ethylbenzothiazoline-6-sulphonic acid)
ACAE	Acarbose equivalent
AChE	Acetylcholinesterase
ANN	Artificial neural network
BChE	Butyrylcholinesterase
CE	Catechin equivalents
CUPRAC	Cupric reducing antioxidant capacity
DPPH	2.2-diphenyl-1-picryl-hydrazyl-hydrate
EDTA	Ethylenediaminetetraacetic acid equivalents
FRAP	Ferric reducing antioxidant power
GAE	Gallic acid equivalents
GALAE	Galantamine equivalents
HCA	Hierarchical cluster analysis
KAE	Kojic acid equivalents
MH	Metal chelating assay
MLP	Multi-layer perceptron
MPE	Mean percentage error
PCA	Principal Component Analysis
PM	Phosphomolybdenum assay
RMSE	Root mean square error
RE	Rutin equivalents
RF	Random forests
SSE	Sum of square error
SVM	Support vector machines
TE	Trolox equivalents
TF	Total flavonoids content
TP	Total phenolics content
UAE	Ultrasound-assisted extraction
UVA	Ultraviolet A
